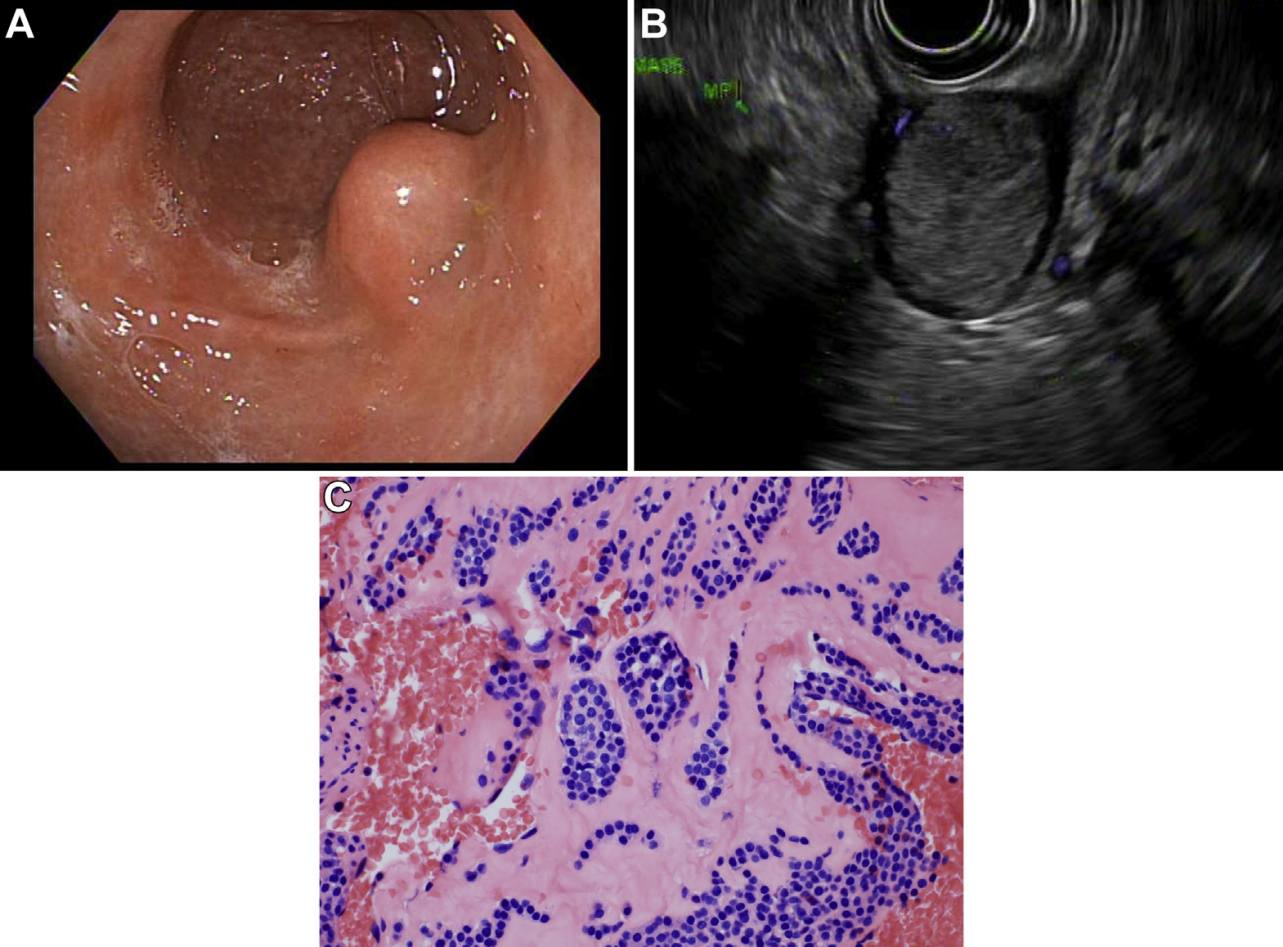# The Onus of the Glomus: Gastric Glomus Tumor

**DOI:** 10.1016/j.gastha.2021.09.008

**Published:** 2022-02-03

**Authors:** Echko Holman, Nicholas M. McDonald, Mohammad Bilal

**Affiliations:** 1 Department of Internal Medicine, University of Minnesota Medical Center, Minneapolis, Minnesota; 2 Division of Gastroenterology, Hepatology, and Nutrition, University of Minnesota, Minneapolis, Minnesota; 3 Division of Gastroenterology and Hepatology, Minneapolis Veterans Affairs Health Care System, Minneapolis, Minnesota

A 58-year-old man was incidentally found to have a gastric mass on computed tomography (CT) scan of the abdomen for an episode of epigastric abdominal pain. The CT scan revealed a 25 × 19 × 20 mm intraluminal density along the lesser curvature of the distal stomach. An upper endoscopy was performed, and a subepithelial lesion was seen in the gastric antrum (Figure A). Endoscopic ultrasound revealed a hypoechoic (30 × 20 mm) well-defined oval subepithelial lesion appearing to originate from muscularis propria (Figure B). Endoscopic ultrasound-guided fine needle biopsy was performed, and pathology revealed a neoplasm composed of uniform, round cells, positive for smooth muscle actin, synaptophysin, and DOG1, consistent with glomus tumor (Figure C). Gastric glomus tumors are indistinguishable from other subepithelial lesions on both CT scan and endoscopic ultrasound. Immunohistochemical testing, histopathology, and molecular testing are essential to establish the diagnosis. As potential malignant transformation cannot be reliably prognosticated on immunohistopathology and given prior rare reports of metastasis, surgery can be considered. In this case, the patient was evaluated by general surgery and surgery is planned. It is important to include glomus tumor on the differential diagnosis of gastric subepithelial lesions as disease recurrence is extremely rare after surgical excision.

**Figure:**